# Supplementary material for: The ex planta signal activity of a Medicago ribosomal uL2 protein suggests a moonlighting role in controlling secondary rhizobial infection
Source: PLoS One. 2020 Oct 1;15(10):e0235446. doi: 10.1371/journal.pone.0235446 (PMC7529298; doi:10.1371/journal.pone.0235446)
Supplement: S1 Fig — Panel A: Signal 1 purification from E. coli DH5a crude extracts. From top to bottom: Purification chart flow, activity assay and SDS-PAGE analysis of fractions eluted from the SP column (B6 corresponds to the 0.3M NaCl fraction). Panel B: Signal 1 purification from a E. coli strain overexpressing a TopA-his tagged protein. From top to bottom: purification flowchart, activity and SDS-PAGE analysis of fractions eluted from the heparin column (G12 corresponds to the 0.32M salt fraction). See methods for details. The black and grey arrowheads points to the RPuL2 and the TopA-His proteins, respectively. The black arrowhead band was excised and identified by MS analysis as being RPuL2 (RplB) (see S1 Table). (PPTX) [file pone.0235446.s001.pptx]

## Slide 1
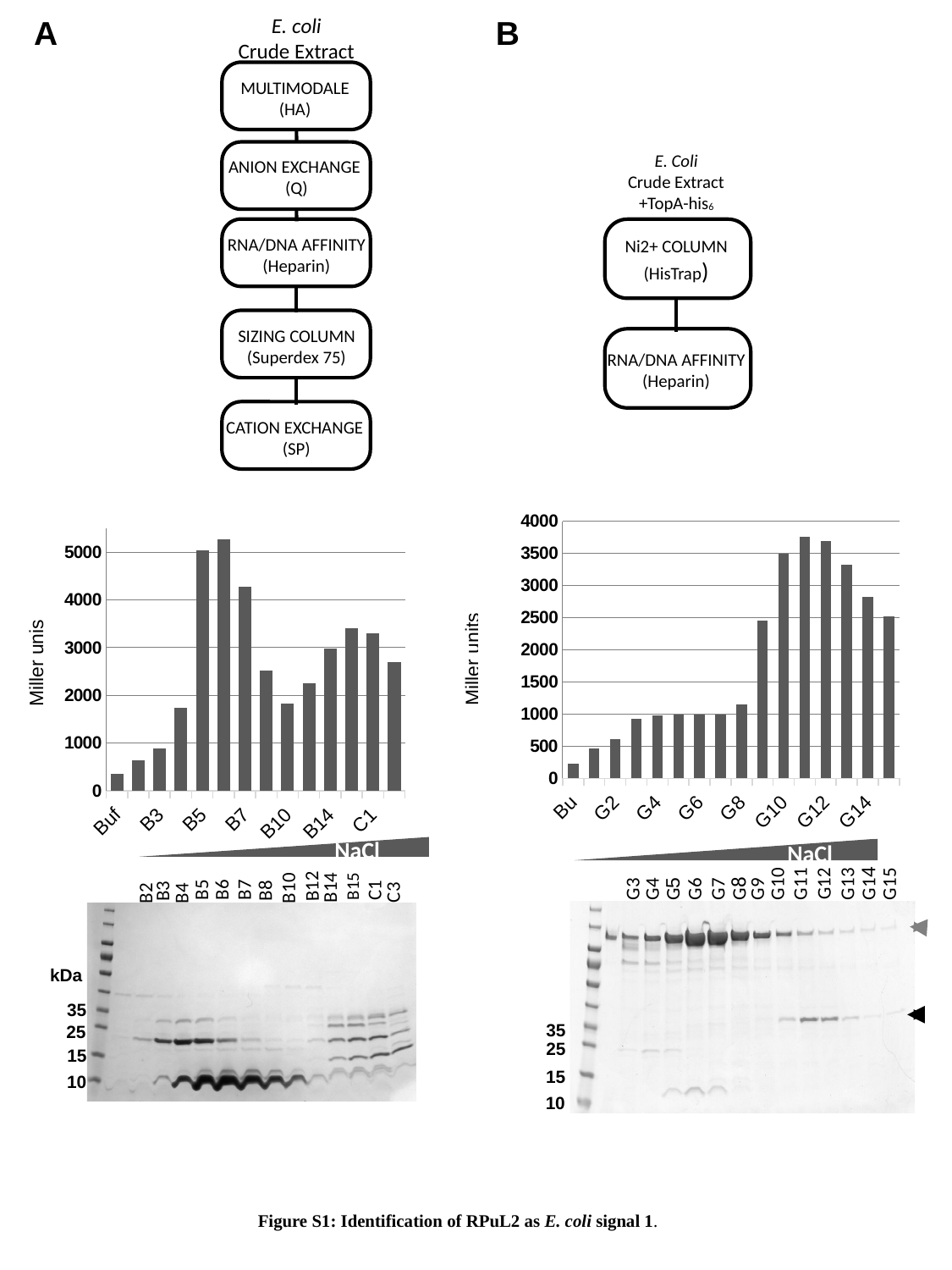

A
B
### Chart
| Category | |
|---|---|
| Buffer | 351.20038488256426 |
| B2 | 640.6942022109776 |
| B3 | 883.9627699400535 |
| B4 | 1736.5931557416811 |
| B5 | 5037.5199558527 |
| B6 | 5276.280127426302 |
| B7 | 4282.5432508869835 |
| B8 | 2526.2194022087565 |
| B10 | 1823.2211223357042 |
| B12 | 2256.614096185972 |
| B14 | 2987.8330091394205 |
| B15 | 3409.8561036485926 |
| C1 | 3302.99354665391 |
| C3 | 2701.343486723071 |NaCl
NaCl gradient
G5
G10
G8
G13
G7
G14
G6
G12
G15
G11
G3
G9
G4
35
25
15
10
B15
B3
B6
B8
B5
B10
B7
B12
C1
B14
B2
B4
C3
35
25
15
10
Miller units
Miller unis
E. coli
Crude Extract
MULTIMODALE
(HA)
ANION EXCHANGE
(Q)
RNA/DNA AFFINITY
(Heparin)
SIZING COLUMN
(Superdex 75)
CATION EXCHANGE
(SP)
E. Coli
Crude Extract
+TopA-his6
Ni2+ COLUMN
(HisTrap)
RNA/DNA AFFINITY
(Heparin)
### Chart
| Category | |
|---|---|
| Buffer | 224.6376811594203 |
| G1 | 467.62589928057525 |
| G2 | 607.4074074074073 |
| G3 | 924.9999999999999 |
| G4 | 979.7979797979796 |
| G5 | 997.4160206718344 |
| G6 | 997.5124378109431 |
| G7 | 989.583333333336 |
| G8 | 1157.181571815718 |
| G9 | 2460.6060606060587 |
| G10 | 3488.8888888888837 |
| G11 | 3752.380952380961 |
| G12 | 3697.777777777778 |
| G13 | 3323.4567901234564 |
| G14 | 2821.0526315789475 |
| G15 | 2523.404255319149 |kDa
Figure S1: Identification of RPuL2 as E. coli signal 1.
