# Supplementary material for: The ex planta signal activity of a Medicago ribosomal uL2 protein suggests a moonlighting role in controlling secondary rhizobial infection
Source: PLoS One. 2020 Oct 1;15(10):e0235446. doi: 10.1371/journal.pone.0235446 (PMC7529298; doi:10.1371/journal.pone.0235446)
Supplement: S2 Fig — Panel A: Synthetic Strep-Tag® peptide (SAWSHPQFEK; 60 μg) activity. B control buffer. Panel B: Activity of a purified SMc02178-Strep-Tag® protein compared to RPuL2 activity. 1μg of each protein was assayed. Panel C: Activity and SDS-PAGE analysis of protein fractions in a mock purification (empty vector) assay on a Strep-Tactin® resin. B control buffer. CE crude extract. MW molecular weight ladder. F flow-through of the Strep-Tactin® resin. W last wash of the Strep-Tactin® resin. E1-E6 elution fractions. (PPTX) [file pone.0235446.s002.pptx]

## Slide 1
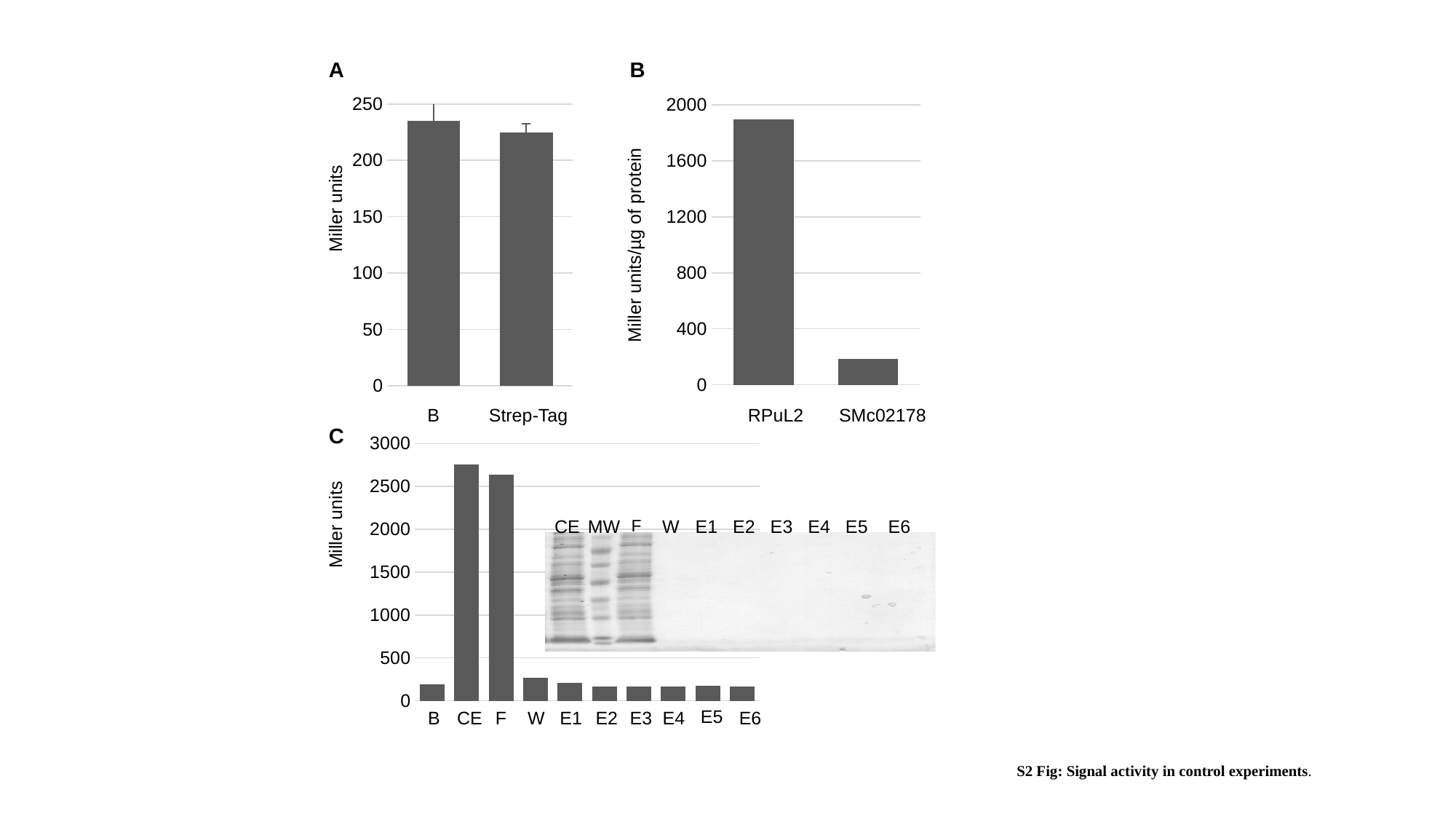

A
B
### Chart
| Category | |
|---|---|
| Buffer | 234.80220158238777 |
| Peptide Strep-tag | 224.6174147489938 |
### Chart
| Category | |
|---|---|
| L2-Streptag | 1897.727272727276 |
| SMc02178-Streptag | 184.4272727272727 |Miller units
Miller units/µg of protein
B
Strep-Tag
RPuL2
 SMc02178
C
### Chart
| Category | |
|---|---|
| Control buffer | 187.9 |
| Crude extract | 2758.8 |
| Flowthrough | 2636.9 |
| Last wash step | 268.3 |
| E1 | 209.7 |
| E2 | 163.9 |
| E3 | 167.4 |
| E4 | 161.9 |
| E5 | 172.1 |
| E6 | 163.6 |E5
B
CE
F
 W
 E1
 E2
 E3
 E4
E6
Miller units
CE
MW
F
 W
E1 E2 E3 E4 E5 E6
S2 Fig: Signal activity in control experiments.
