# Supplementary material for: The ex planta signal activity of a Medicago ribosomal uL2 protein suggests a moonlighting role in controlling secondary rhizobial infection
Source: PLoS One. 2020 Oct 1;15(10):e0235446. doi: 10.1371/journal.pone.0235446 (PMC7529298; doi:10.1371/journal.pone.0235446)
Supplement: S3 Fig — Specific activities of purified amino-terminal (1–121) and carboxy-terminal (122–273) moieties of E. coli RPuL2 (10-fold dilution of the main elution fraction from the Strep-Tactin® column. Both proteins were strep-tagged at the carboxy-terminal end. (PPTX) [file pone.0235446.s003.pptx]

## Slide 1
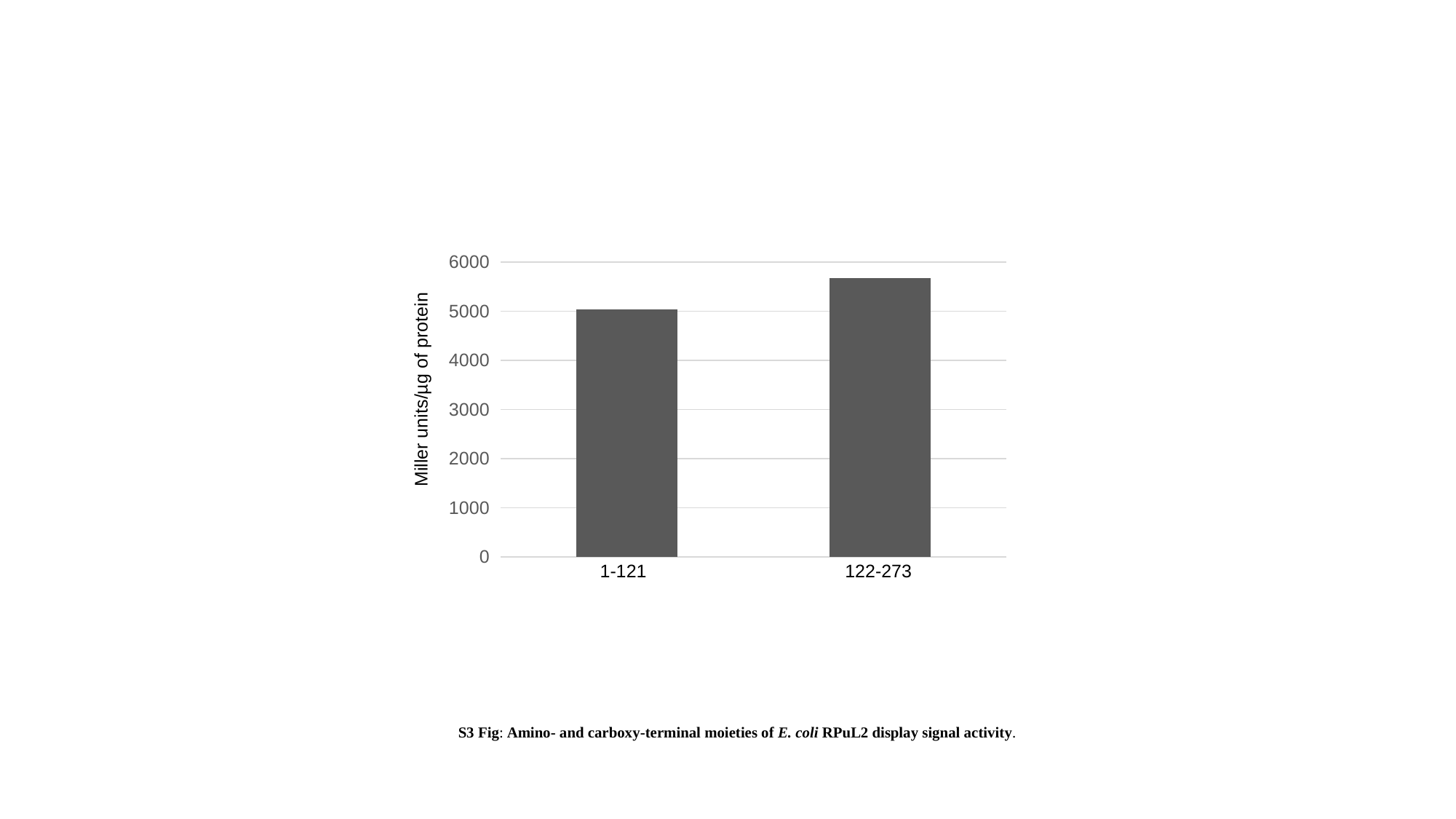

### Chart
| Category | |
|---|---|
| RplB 1-121 | 5028.58 |
| RplB 122-273 | 5676.605504587154 |Miller units/µg of protein
1-121
122-273
S3 Fig: Amino- and carboxy-terminal moieties of E. coli RPuL2 display signal activity.
