# Supplementary material for: The ex planta signal activity of a Medicago ribosomal uL2 protein suggests a moonlighting role in controlling secondary rhizobial infection
Source: PLoS One. 2020 Oct 1;15(10):e0235446. doi: 10.1371/journal.pone.0235446 (PMC7529298; doi:10.1371/journal.pone.0235446)
Supplement: S4 Fig — Signal activity of the Input (I) and flow-through (F) fractions of a fiberglass column. B buffer control. P-value 0.0078, t-test, n = 5. The right panel features a representative western blot using anti-human RPL8 protein antibodies. Please note that the human anti-RPL8 antibodies cannot detect low amounts of heterologous RPuL2 proteins. (PPTX) [file pone.0235446.s004.pptx]

## Slide 1
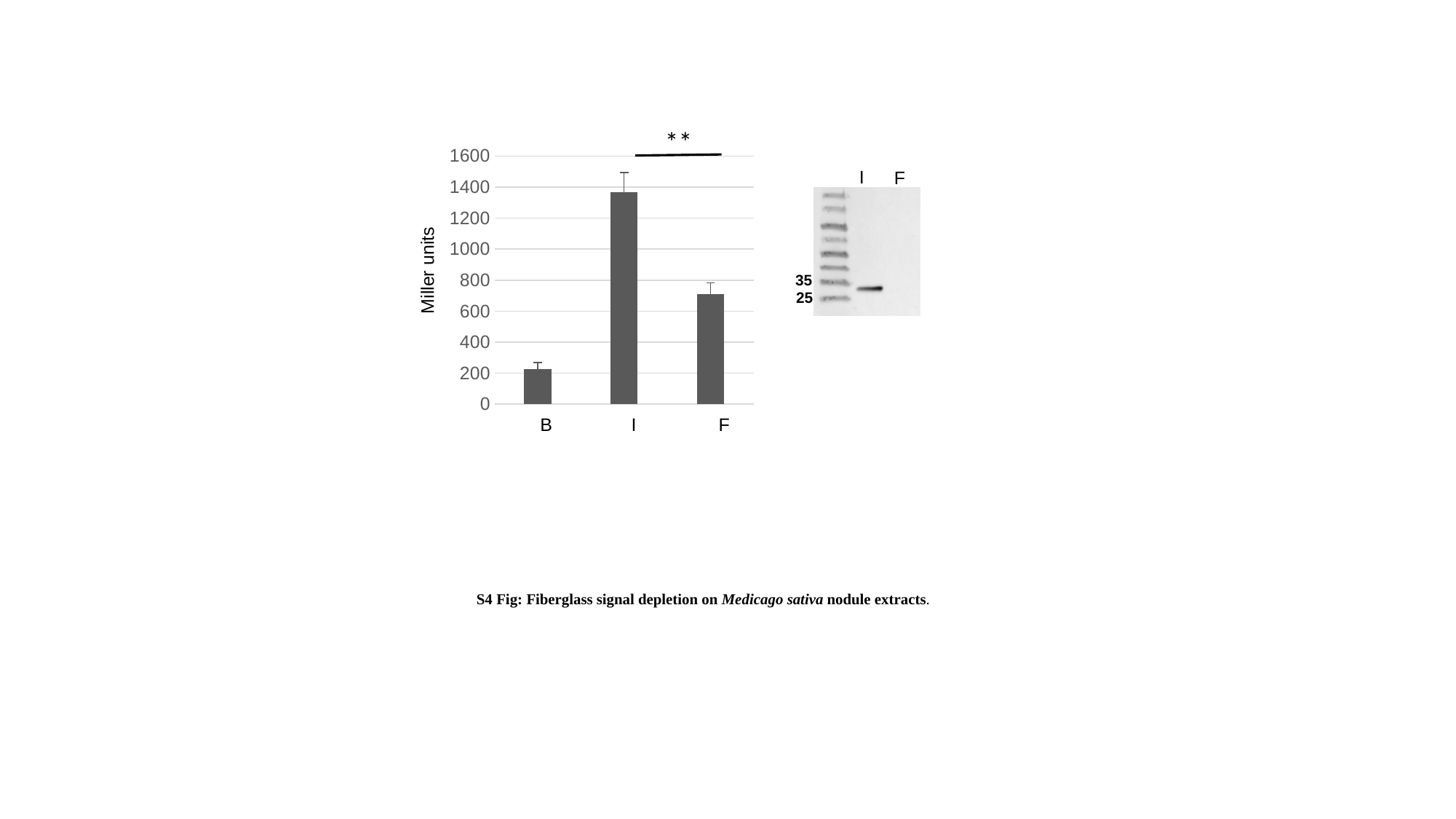

**
### Chart
| Category | |
|---|---|I
F
35
25
Miller units
B
I
F
S4 Fig: Fiberglass signal depletion on Medicago sativa nodule extracts.
