# Supplementary material for: The ex planta signal activity of a Medicago ribosomal uL2 protein suggests a moonlighting role in controlling secondary rhizobial infection
Source: PLoS One. 2020 Oct 1;15(10):e0235446. doi: 10.1371/journal.pone.0235446 (PMC7529298; doi:10.1371/journal.pone.0235446)
Supplement: S6 Fig — Panel A: RNAse treatment of Mt A17 and Mtnf-ya1 nodule extracts n = 3. Panel B: Effect of RNASe inhibitors (P-value = 0.046, t-test, n = 3). 25 mg of nodules fresh weight were used per assay. (PPTX) [file pone.0235446.s006.pptx]

## Slide 1
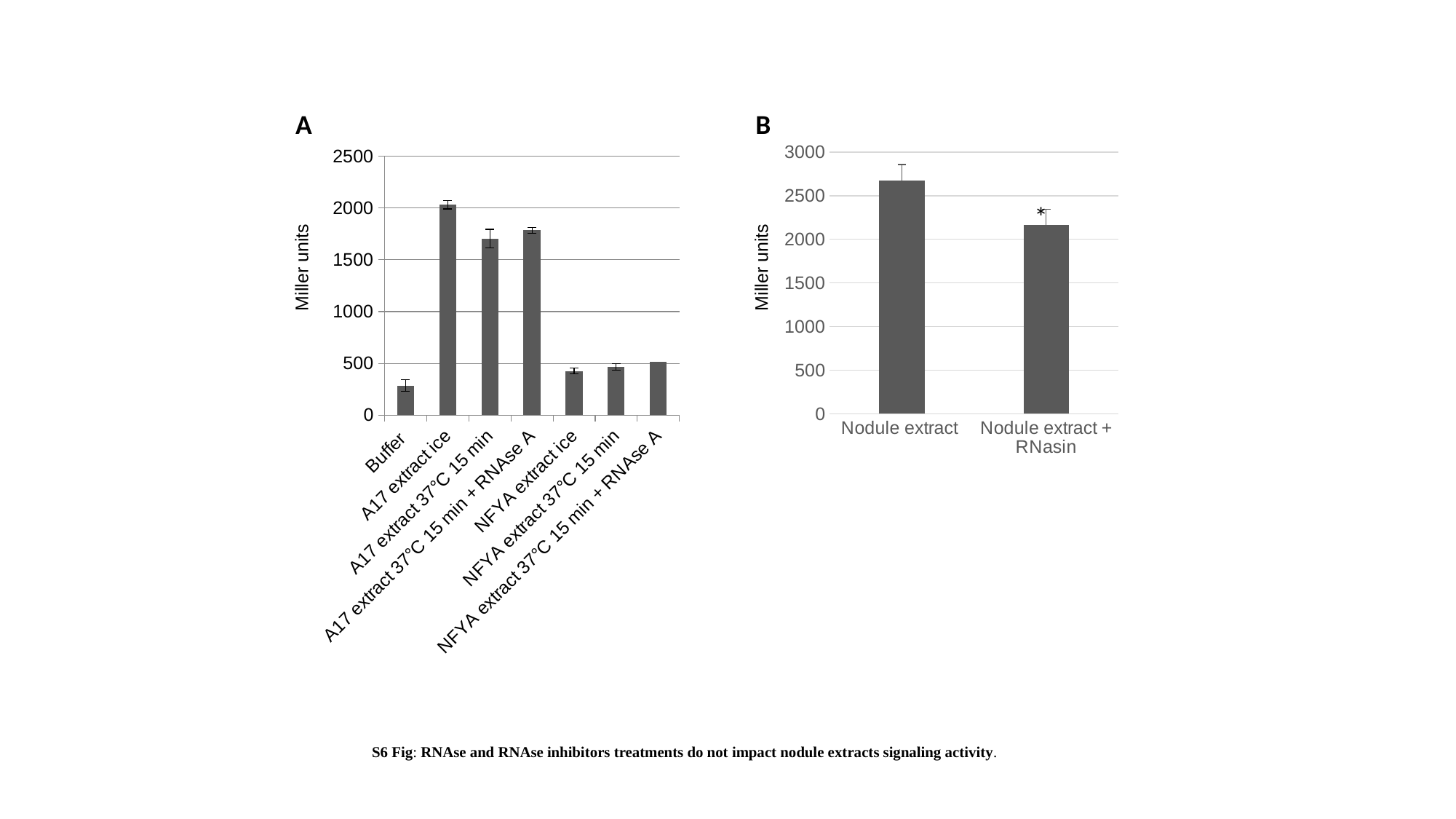

A
B
### Chart
| Category | |
|---|---|
| Buffer | 284.6 |
| A17 extract ice | 2029.1099048221222 |
| A17 extract 37°C 15 min | 1703.8716136182481 |
| A17 extract 37°C 15 min + RNAse A | 1783.5329784087546 |
| NFYA extract ice | 426.6178804443661 |
| NFYA extract 37°C 15 min | 464.2857142857143 |
| NFYA extract 37°C 15 min + RNAse A | 515.9637613245861 |
### Chart
| Category | |
|---|---|
| Nodule extract | 2674.512149081789 |
| Nodule extract + RNasin | 2164.6462699094277 |*
Miller units
Miller units
S6 Fig: RNAse and RNAse inhibitors treatments do not impact nodule extracts signaling activity.
