# Supplementary material for: The ex planta signal activity of a Medicago ribosomal uL2 protein suggests a moonlighting role in controlling secondary rhizobial infection
Source: PLoS One. 2020 Oct 1;15(10):e0235446. doi: 10.1371/journal.pone.0235446 (PMC7529298; doi:10.1371/journal.pone.0235446)
Supplement: S7 Fig — Panel A: Charge distribution of selected ribosomal proteins according to Pepcal (https://pepcalc.com). Color code for amino acids: red acidic, green aromatic, cyan basic, dark green polar. Top line is hydrophilic, bottom line is hydrophobic. Panel B visualisation of acidic residues (DE, underlined yellow) in the surface exposed (blue) and inner loops (green) of the beta-barrel portion of NsrA (amino acids 600–1200). TM regions are shown red. Loop prediction was done using the Pred-TMBB software. (PPTX) [file pone.0235446.s007.pptx]

## Slide 1
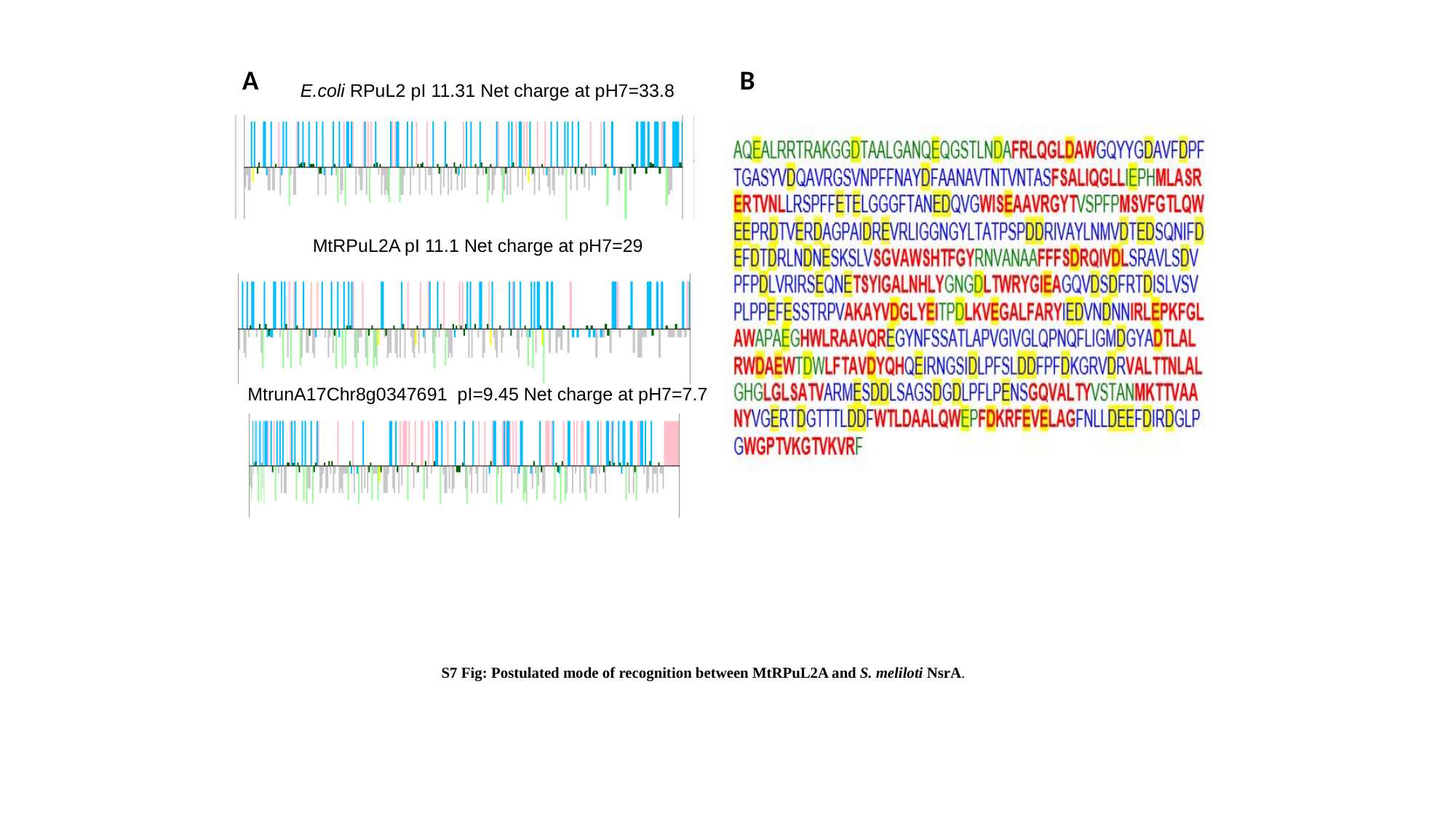

A
B
E.coli RPuL2 pI 11.31 Net charge at pH7=33.8
MtRPuL2A pI 11.1 Net charge at pH7=29
MtrunA17Chr8g0347691 pI=9.45 Net charge at pH7=7.7
S7 Fig: Postulated mode of recognition between MtRPuL2A and S. meliloti NsrA.
