# Supplementary material for: The ex planta signal activity of a Medicago ribosomal uL2 protein suggests a moonlighting role in controlling secondary rhizobial infection
Source: PLoS One. 2020 Oct 1;15(10):e0235446. doi: 10.1371/journal.pone.0235446 (PMC7529298; doi:10.1371/journal.pone.0235446)
Supplement: S1 Raw images — (PDF) [file pone.0235446.s008.pdf]

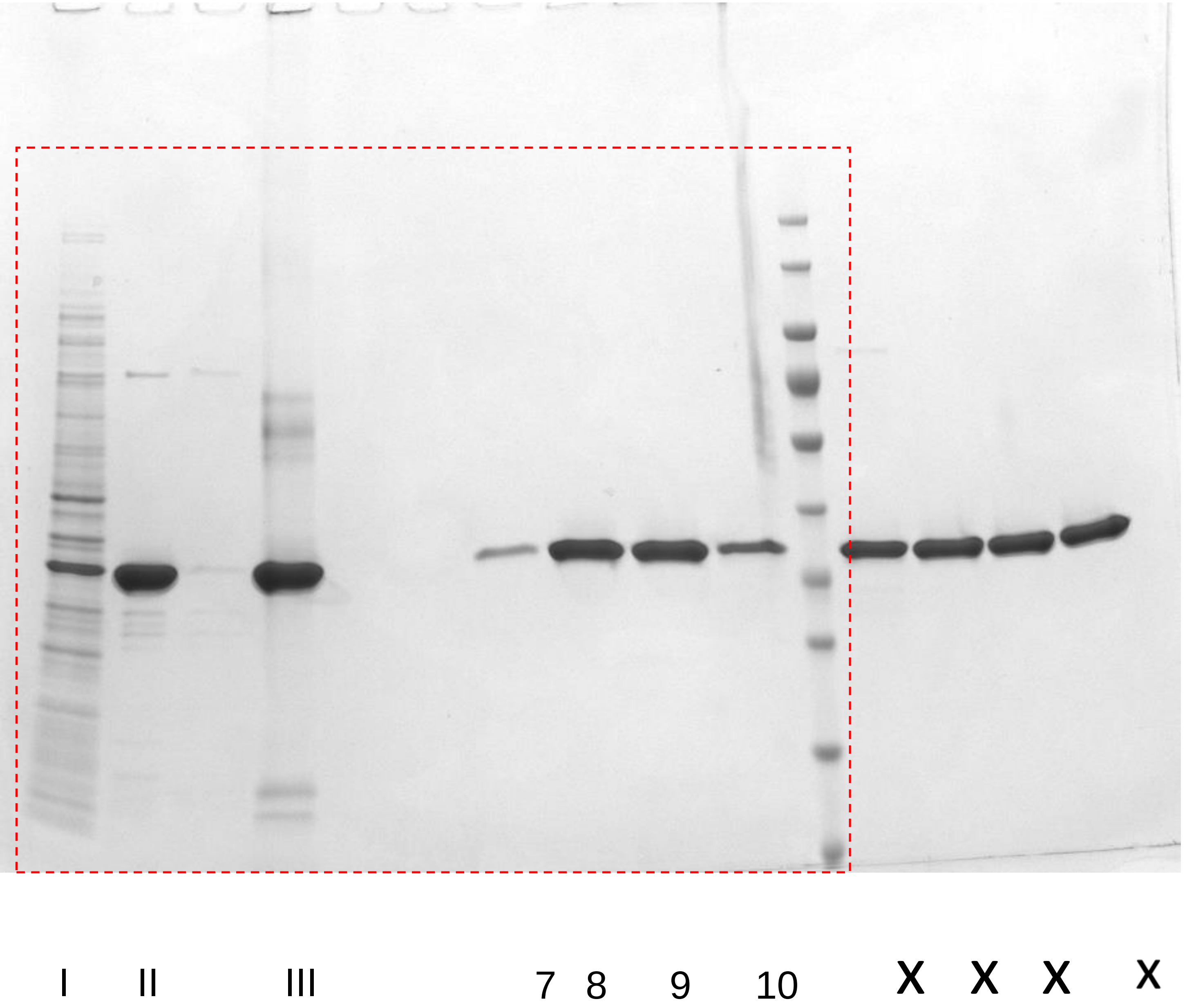

Fig. 2A

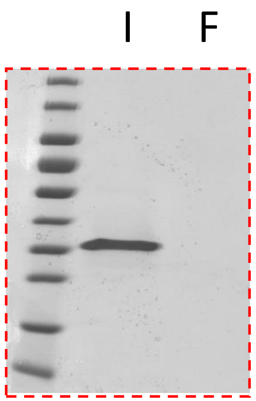

Fig 2D

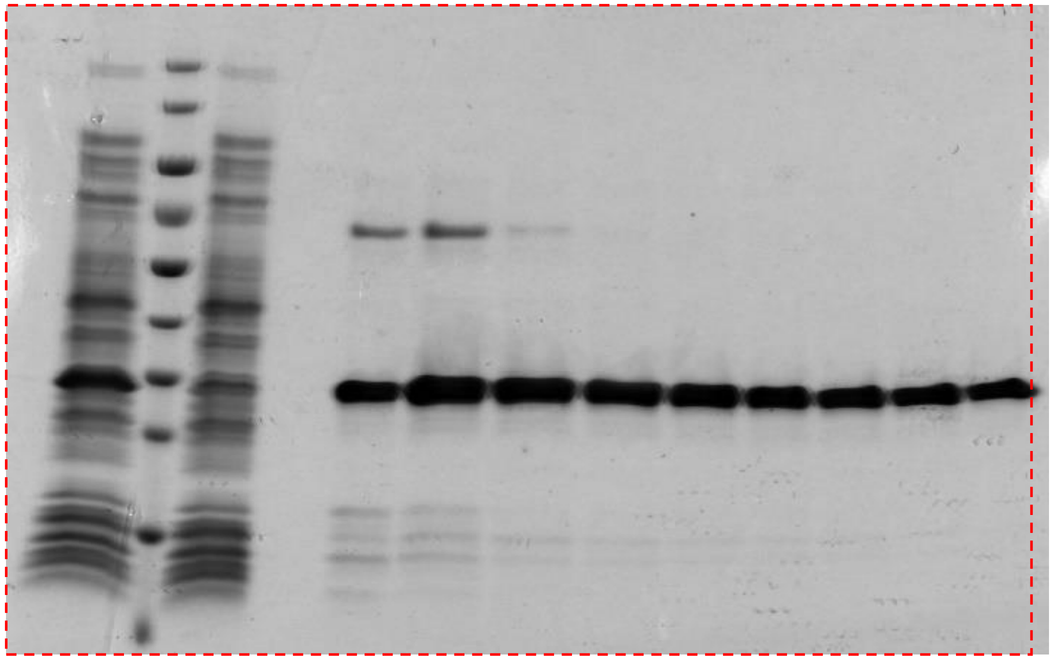

I II III IV 1 2 3 4 5 6 7 8 9

V

Fig 4A raw

Fig4C raw

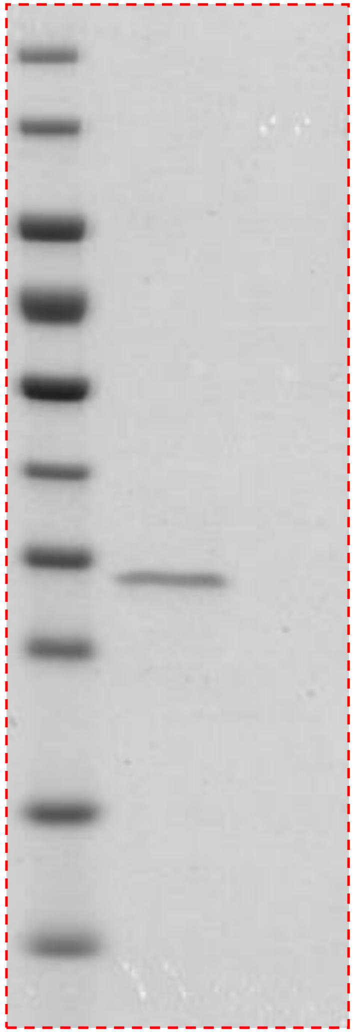

I F

MtRPuL2A

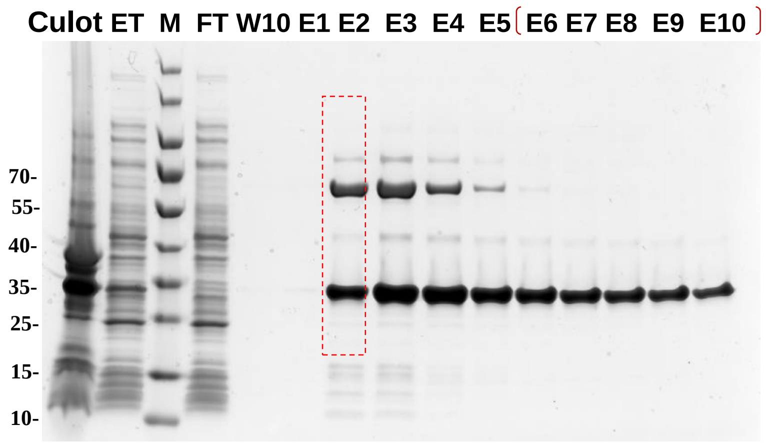

MtrunA17Chr8g0347691

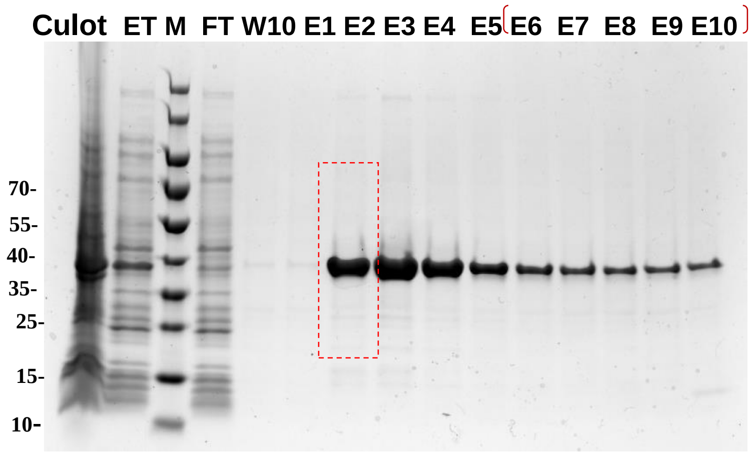

Fig5 raw insert

Purification of strep-tagged proteins on strep-Tactin columns: Culot (pellet), ET (crude extract), M molecular weight marker, W10 last wash of strep-tactin column, E1-E10 elution fractions with 5mM desthiobiotine (see material and methods for details)

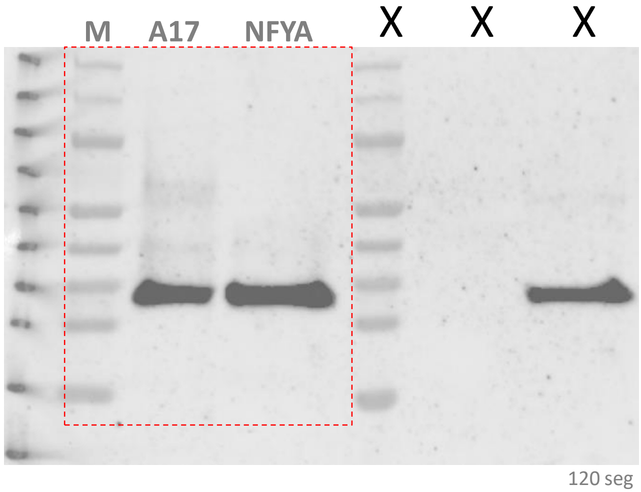
M
A17
NFYA
X
X
X
120 seg

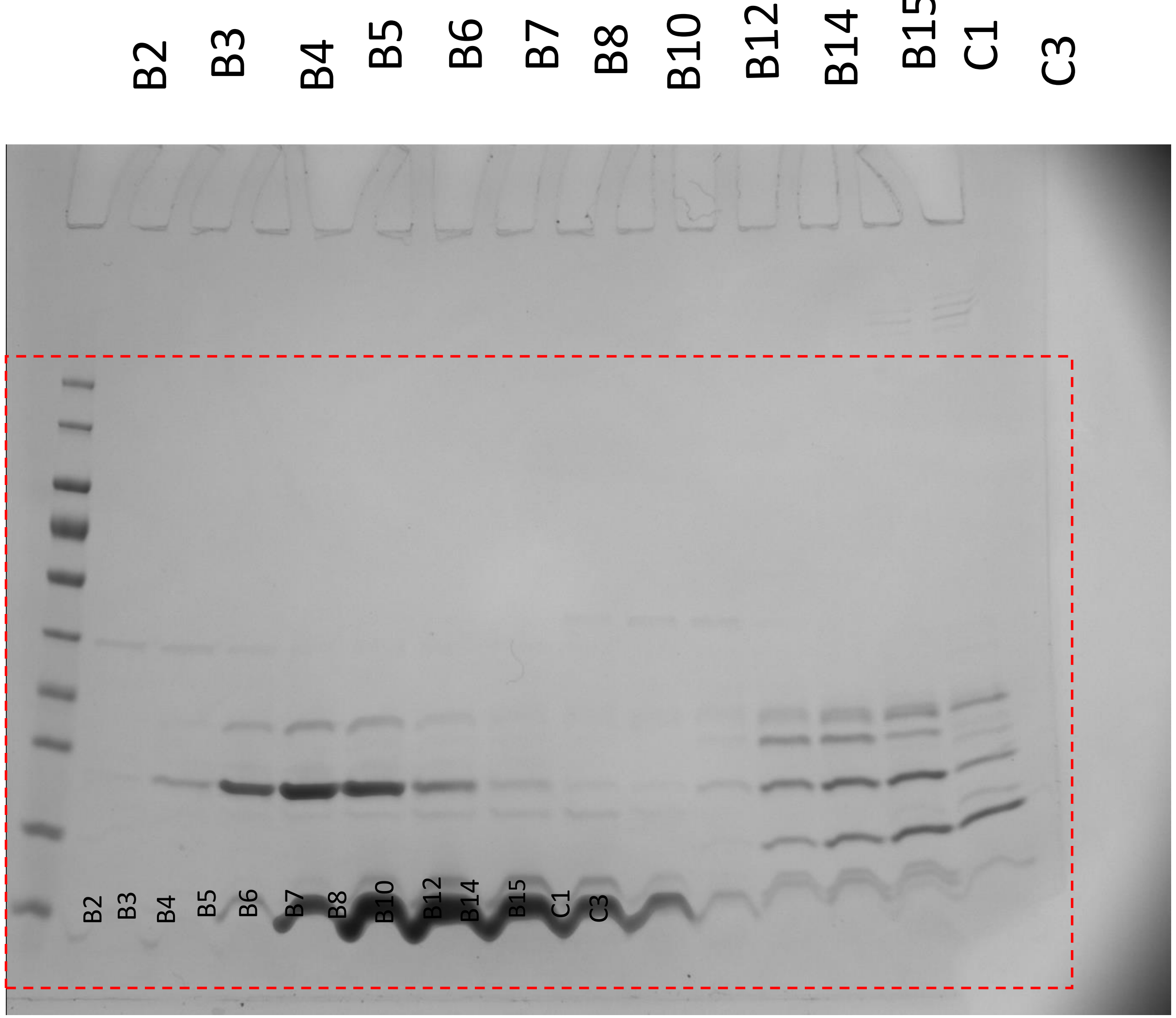

S1 Fig panel A

Signal 1 purification from *E. coli* DH5a crude extracts. SDS-PAGE analysis of fractions eluted from the SP column with a NaCl gradient. See legend of S1 Fig for details.

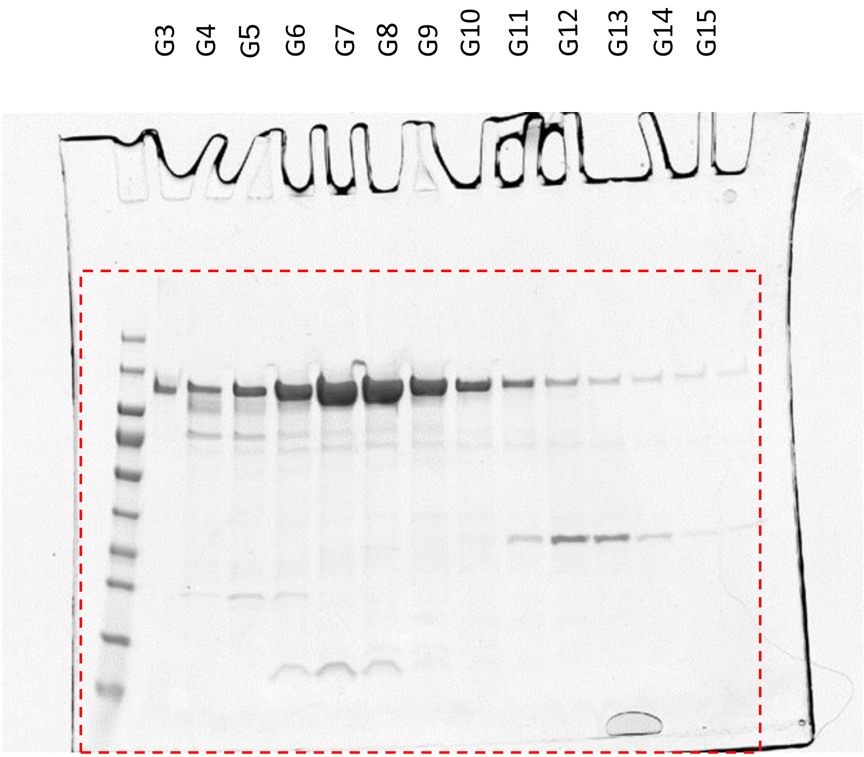

S1B Fig raw

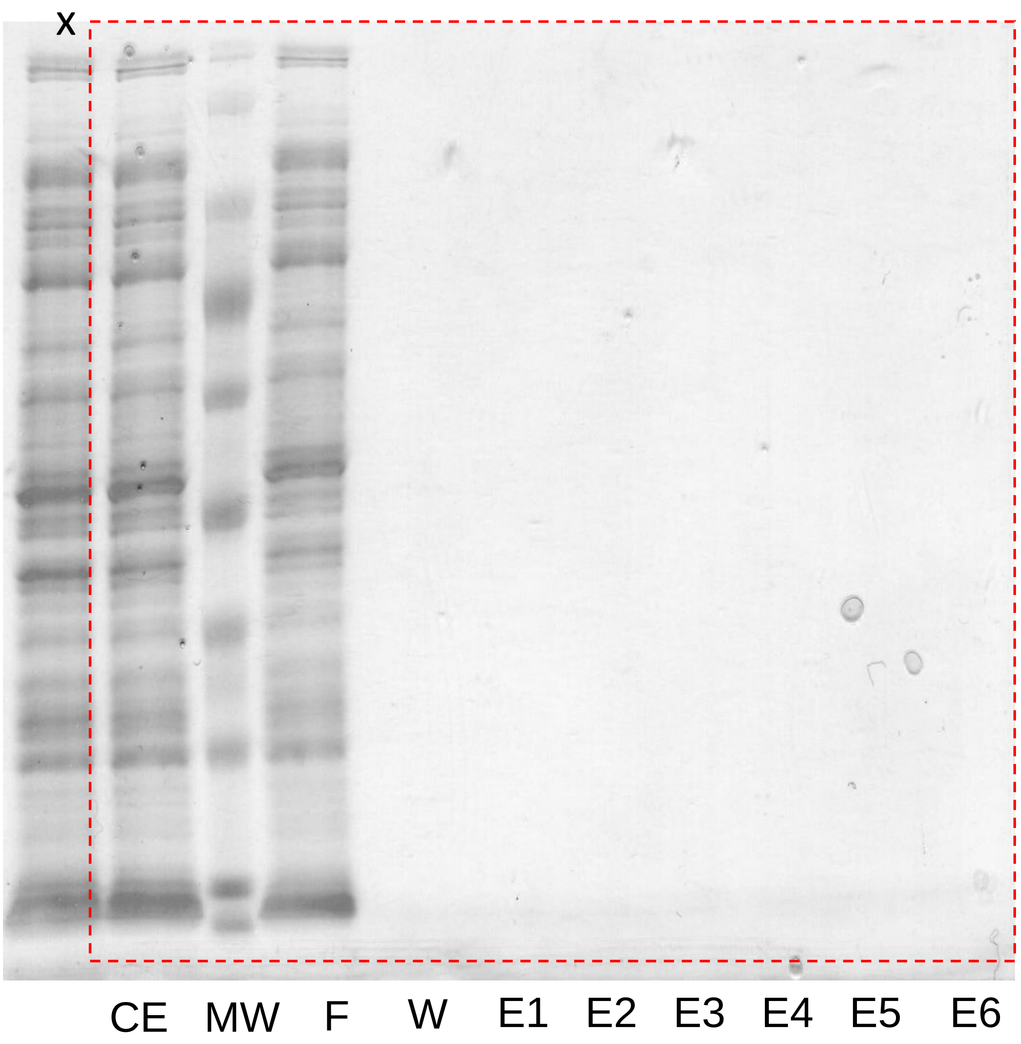

S2 Fig raw

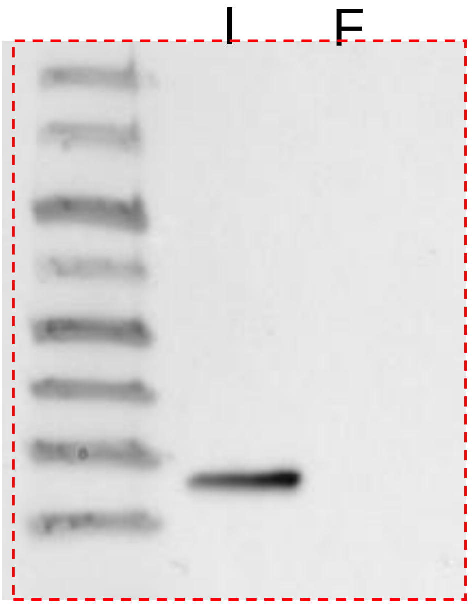

S4 Fig Raw
