# Supplementary material for: The ex planta signal activity of a Medicago ribosomal uL2 protein suggests a moonlighting role in controlling secondary rhizobial infection
Source: PLoS One. 2020 Oct 1;15(10):e0235446. doi: 10.1371/journal.pone.0235446 (PMC7529298; doi:10.1371/journal.pone.0235446)
Supplement: S4 Table — (DOCX) [file pone.0235446.s012.docx]

**S4 Table : Bacterial strains and plasmids used in this study.**

| **Strain or plasmid** | **Description** | **Source or reference** |
| --- | --- | --- |
| *Escherichia coli* |  |  |
| DH5α | *E. coli* *fhuA2 Δ(argF-lacZ)U169 phoA glnV44 φ80 Δ(lacZ)M15 gyrA96 recA1 relA1 endA1 thi-1 hsdR17* | Bethesda Research Laboratories |
| BL21-Rosetta (DE3) pLysS | F–, *omp*T, *hsd*S_B_ (r_B_–,m_B_ ), *dcm*, *gal*, λ(DE3), pLysS, Chloramphenicol^R^ | Novagen |
| *Sinorhizobium meliloti* |  |  |
| Rm1021 | Streptomycine^R^ derivative of *S. meliloti* strain SU47 | Meade et al., 1982 |
| GMI12052 | Rm1021 strain overexpression *nsrA*, 1021(pGMI50333, pGD2178) Streptomycine^R^ , Gentamycine^R^, Tetracycline^R^ | Garnerone et al., 2018 |
| GMI12071 | Rm1021 *cyaK* mutant overexpressing *nsrA*, *cyaK* (pGMI50333, pGD2178) | This work |
| \| GMI12072 \|  \|  \| \| --- \| --- \| --- \| | Rm1021 Δ*nsrA* Str^r^ (pGD2178) | \|  \|  \| This work \| \| --- \| --- \| --- \| |
| *Saccharomyces cerevisiae* BY4741 | *MATα his3Δ1 leu2Δ0 met15Δ0 ura3Δ0* | Dr [JM](https://www.yeastgenome.org/reference/S000041186) Francois INSA Toulouse |
| Plasmids |  |  |
| pBlueScript II SK(+) | General cloning vector | Agilent Technologies |
| pCDFDuet-1 | Expression vector | Novagen |
| pCDFDuet-1 His_6_-TopA | Plasmid for the expression of an amino-terminal His_6_ -tagged TopA | This work |
| pCDFDuet-1-RPuL2StrepTag | Plasmid for the expression of a carboxy-terminal Strep-tagged *E. coli* RPuL2 | This work |
| pCDFDuet-1-121-L2StrepTag | Plasmid for the expression of a carboxy-terminal Strep-tagged amino-terminal fragment (aa 1-121) of *E. coli* RPuL2 | This work |
| pCDFDuet-1-122-273L2StrepTag | Plasmid for the expression of a carboxy-terminal Strep-tagged carboxy-terminal fragment (aa 122-273) of *E. coli* RPuL2 | This work |
| pCDFDuet-1-MtRPuL2A-Streptag | Plasmid for the expression of a carboxy-terminal Strep-tagged MtRPuL2A | This work |
| pCDFDuet-1-MtrunA17Chr8g0347691 -Streptag | Plasmid for the expression of a carboxy-terminal Strep-tagged MtrunA17Chr8g0347691 | This work |
| pGD2178 | pGD926 containing the *smc02178* promoter region fused to *lacZ*; Tet^r^ | Tian et al. 2012 |
| pRK600 | Helper conjugative plasmid, ColE1 replicon with RK2 transfer region , Chl^R^ | Finan et al, 1986 |

References:

Meade HM, Long SR, Ruvkun GB, Brown SE, Ausubel FM. Physical and genetic characterization of symbiotic and auxotrophic mutants of Rhizobium meliloti induced by transposon Tn5 mutagenesis. Journal of Bacteriology. 1982; 149: 114-122.

Garnerone A-M, Sorroche F, Zou L, Mathieu-Demaziere C, Tian CF, Masson-Boivin C, et al. NsrA, a Predicted beta-Barrel Outer Membrane Protein Involved in Plant Signal Perception and the Control of Secondary Infection in Sinorhizobium meliloti. Journal of Bacteriology. 2018. DOI: [10.1128/JB.00019-18.](https://doi.org/10.1128/jb.00019-18)

Tian CF, Garnerone AM, Mathieu-Demaziere C, Masson-Boivin C, Batut J. Plant-activated bacterial receptor adenylate cyclases modulate epidermal infection in the Sinorhizobium meliloti-Medicago symbiosis. Proceedings of the National Academy of Sciences of the United States of America. 2012;109(17):6751-6.

Finan TM, Kunkel B, De Vos GF, Signer ER. Second symbiotic megaplasmid in Rhizobium meliloti carrying exopolysaccharide and thiamine synthesis genes. Journal of Bacteriology. 1986;167:66-72.
