# Supplementary material for: The ex planta signal activity of a Medicago ribosomal uL2 protein suggests a moonlighting role in controlling secondary rhizobial infection
Source: PLoS One. 2020 Oct 1;15(10):e0235446. doi: 10.1371/journal.pone.0235446 (PMC7529298; doi:10.1371/journal.pone.0235446)
Supplement: S5 Table — (DOCX) [file pone.0235446.s013.docx]

**S5 Table : Oligonucleotide primers used in this study**

| Primer Designation | Sequence (5’-3’) |
| --- | --- |
| topA/TOPRIM-F | gagctccGGTAAAGCTCTTGTCATCGTTG |
| topA/ZF+ribb-R | AAGCTTTTATTTTTTTCCTTCAACCCATTTG |
| L2BglII-Fw | GGTATAGATCTAATGGCAGTTGTTAAATG |
| L2-StrepTAG-rev | GCTGCTCGAGTCATTTTTCGAACTGCGGGTGGCTCCAGCTAGCTAATTTGCTACGGCGAC |
| RevL2-1to121StrepTag | GCTGCTCGAGTCATTTTTCGAACTGCGGGTGGCTCCAGCTAGCATCAACGCCAGACTGAATCTGG |
| L2-122-BglII-Fw | GGTATAGATCTAATGGCTGCAATCAAACCAGGTAACACC |
| NdeI 2178 Stp | CGCCATATGAAGAAACACGTTGGATCGAGCCTGTATCA |
| XhoI 2178 Stp | CCGCTCGAGTCATTTTTCGAACTGCGGGTGGCTCCAGCTAGCCTGCAATCCGTCCGTG |
