# Supplementary material for: The ex planta signal activity of a Medicago ribosomal uL2 protein suggests a moonlighting role in controlling secondary rhizobial infection
Source: PLoS One. 2020 Oct 1;15(10):e0235446. doi: 10.1371/journal.pone.0235446 (PMC7529298; doi:10.1371/journal.pone.0235446)
Supplement: S5 Fig — Panel A: Affymetrix normalised hybridisation data for a selection of conditions (nodules, leaves, shoots, roots…), extracted from GeneAtlas (https://mtgea.noble.org/v3) showing the strong correlation (0.86) in expression profiles of the two probes. Panel B: Total polyA reads in RNAseq data from 10 day old nodules or roots (from [27]. Panel C: RNAseq counts of ribo-depleted RNA samples from laser microdissected nodule zones (from [27]. (PDF) [file pone.0235446.s005.pdf]

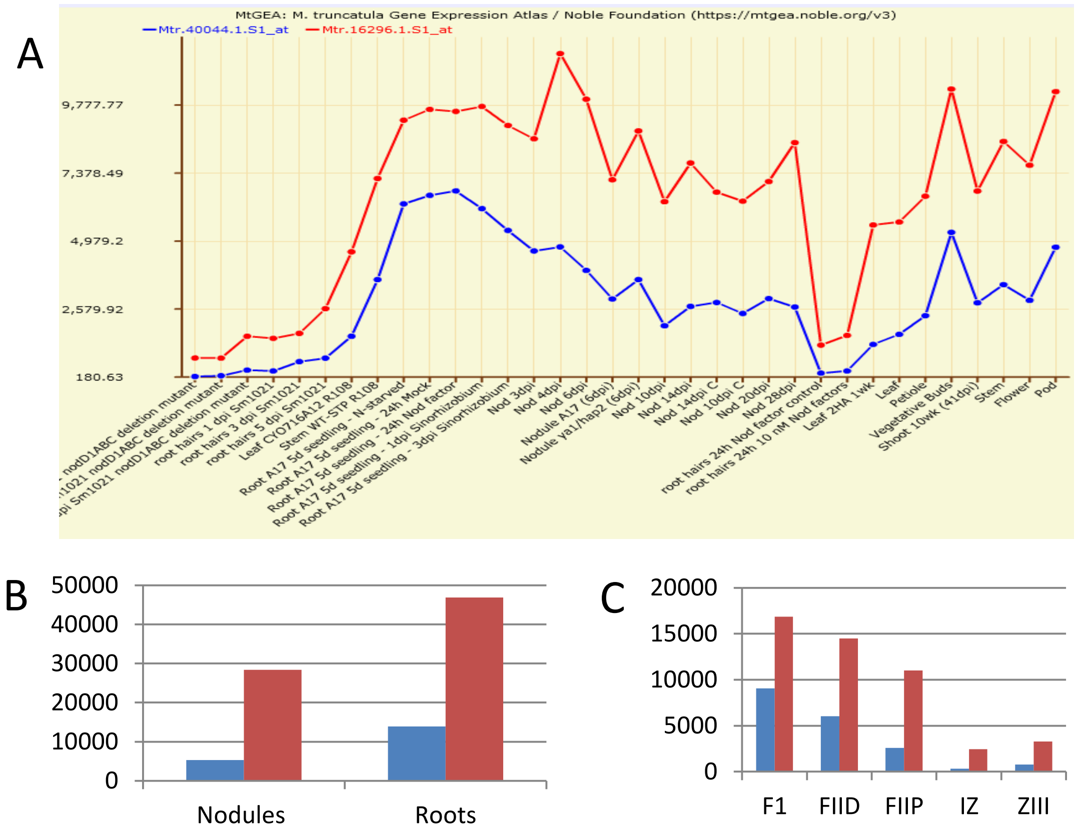

S5 Fig: Expression analysis of *MtRPuL2A* (*MtrunA17Chr7g0247311*, red) and *MtRPuL2B* (*MtrunA17Chr5g0405281*, blue).
