# Supplementary material for: The ex planta signal activity of a Medicago ribosomal uL2 protein suggests a moonlighting role in controlling secondary rhizobial infection
Source: PLoS One. 2020 Oct 1;15(10):e0235446. doi: 10.1371/journal.pone.0235446 (PMC7529298; doi:10.1371/journal.pone.0235446)
Supplement: Attachment — Submitted filename: Responses to Reviewers.docx [file pone.0235446.s014.docx]

Sorroche *et al.*

**Reviewer#1**

1. The units are now specified in the Y axis directly.

Our strategy was to assay the signal activity of as many as possible of the 59 candidate proteins identified in S1 Table. For 30 of them, mutants existed in the Keio collection. For topoisomerase I, no mutant was available (essential gene) but the purified protein existed as a commercial supply (Promega). This part has been rephrased as follows for clarity (l. 146, line numbers s correspond to the marked manuscript). “*Instead, we found that a commercial preparation of E. coli topoisomerase I (TopA, Promega) had a significant signaling activity. The corresponding mutant did not exist in the Keio collection, as expected since top1 is an essential gene in E. coli. To validate this observation, we therefore extensively purified an amino-terminal His6-tagged version of E. coli TopA on Nickel and Heparine-sepharose columns (S1 Fig).”*

2. *E. coli* and *M.truncatula* RPuL2, as orthologous proteins (Fig 3A), display sequence similarity that reflects their phylogenetic relatedness but probably says nothing on the molecular determinants of signal activity. We argue in the paper (l. 162-168 and S3 Fig) that activity does not rely on a specific motif /domain of the RPuL2 proteins. Instead, we propose (l. 396-403 and S7 Fig) that basic residues/patches at the surface of both *E. coli* and *Medicago* RPuL2 proteins may account for activity.

The fact that the *E. coli* and *Medicago* proteins were (over)produced in a homologous and heterologous genetic context (*E.coli),* respectively*,* may account for the difference in activity/solubility *eg* as a folding or post-translational modification issue.

3. Fig 2, 4, 5: Error bars have been added to Fig 2C, Fig 4 (a new set of data has been included) and Fig 5 as well as in SI Figures, whenever possible.

Additional comments:

IRLC defined l. 76

L. 256: Rephrased as suggested by reviewer #2: “*As RPs are very basic proteins basic (pI 11.1 for MtRPuL2A/MtRPuL2B vs pI 9.45 for MtrunA17Chr8g0347691), we tested other cationic compounds for signal activity.”*

L. 387: MDM2 corrected

Fig 1B: *cyaK*-dependency of the *Medicago* nodule signal was established in Fig 1A. Fig. 1B aims at testing the effect of proteinase K treatments on signal activity.

Fig 5: Y axis: µM is correct. Since we aim to compare the signal activities of peptides/proteins of very different molecular sizes, µg cannot be used.

Y and X axis now drawn in all Figures

Our understanding is that PLOS One does not use italics for restriction enzymes following Roberts et al. (2003) NAR 31: 1805-1812.

Similarly, it seems that species names should not be italicized in the reference list in PLOS One.

**Reviewer#2**

1. Error bars have been added in Fig 2, 4 and 5. Error bars feature SE, this is now indicated in the figure legends as well as the number of independent assays performed and the statistical test used.
2. L.111 *“Signal 1 is ubiquitous…”* We agree that, at this stage of the paper, ubiquity only relies on *cyaK*-dependency. We changed “ubiquitous” for “widespread” to tune down the assertion. In the Legend of Fig 6 (L. 309) . “*Free RPuL2 is the signal*” was changed for “*Free RPuL2 has signal activity*”.
3. Abstract: We removed the sentence regarding candidate gene expression data as this is not our own experimental data.
4. The rationale for the bioassay is described in the Introduction section with appropriate references (l. 92-97). For clarity, we have now added the following sentence at the beginning of the results section (l. 113-114) “*We used a S. meliloti strain (GMI12052, S4 Table) that overproduces the* NsrA *receptor protein as a reporter strain for signal activity*.” The used strains are now specified in all the figure legends and their genotype described in detail in S4 Table.

We homogenized the wording to “signal activity” throughout the paper, including the title.

5. No, there is no detectable repeat in the *E. coli* RPuL2 protein. Instead, we propose in the discussion section (l. 396-403) and in S7 Fig. that basic residues of the RPuL2 proteins interact with surface-exposed acidic residues of the NsrA-receptor protein.
6. S5 Fig: The figure has now been submitted as a pdf to decrease its size (247Ko).
7. The abstract was modified as follows to clarify this point “*…AOI is initially triggered by so-far unidentified Medicago nodule signals named signal 1 and signal 1’ whose transduction in bacteroids requires the S. meliloti outer-membrane-associated NsrA receptor protein and the cognate inner-membrane-associated adenylate cyclases, CyaK and CyaD1/D2, respectively.”*
8. See below

Specific comments:

l. 39: Changed as suggested “*Biochemical analyses indicated a peptidic nature for signal 1 and, together with proteomic analyses, a universally conserved Medicago ribosomal protein of the uL2 family was identified as a candidate signal 1.”*

l. 42 “*signaling activity ex planta*” was changed for “*signal activity*” for clarity.

l. 49 Edited as suggested.

l. 60 We totally agree. We were actually referring to signal identification in symbiotic systems in general, not in the rhizobium-legume symbiosis specifically. To remove this ambiguity, we changed the sentence to “*Given the critical importance of these signaling events, the molecular identification of the underlying signals is a major challenge in the field*.”

l. 76 corrected

l. 102-103 Changed as follows for clarity “*Purified MtRPuL2A (MtrunA17Chr7g0247311) displays a strong signal activity in our ex planta bioassay that*…”

l. 111. No other bacteria were examined.

l. 126 Added sentence in Fig 1 legend “ *… tested for signal activity in S. meliloti reporter strains GMI12052 (wt, black) and GMI12071 (cyaK,grey).”.*

l. 130 “here” deleted.

l. 136 proteinase K corrected.

l. 184 (legend of Fig. 2). Reporter strains are now specified.

l. 229 changed as suggested “*phylogenetic tree of RPuL2 protein sequences from Medicago truncatula…”*

l. 256 changed as suggested “*As RPs are very basic proteins*…”

l. 269 now explicited “*M. sativa* bacteria-free NAR (Nodulation in the Absence of Rhizobia)…”

l. 280 . No, MtRPuL2 mainly exists as a ribosome–bound protein in nodules. What our results show is that MtRPuL2 *also* exists as a free (non-ribosomal) protein in nodules.

l. 330. The used antibody is now specified in the legend of Fig 7 (l. 342): “*The insert shows a Western blot of M. truncatula* A17 and *Mtnf-ya1* nodules with *a* human *anti-RPL8 (RPuL2) antibody*.” The commercial source of the antibody is specified in the Material and Methods section (l. 582).

l. 338 “ *a blue coloration*” corrected.

l. 341 “*Plants were grown under aeroponic conditions*.” Corrected

l. 348 Corrected as “*Sensing of MtRPuL2A by reporter bacteria has the same genetic requirements as signal 1 sensing*…”

l. 350 corrected as suggested “*the S. meliloti NsrA receptor protein is involved in recognition of two different signals in symbiosis…”*

l. 362: This is a very good point. Yet we think it is unlikely that signaling relates to nodule senescence. Expression of the reporter gene fusion (*smc02178-lacZ)* *in planta* takes place in very young (7dpi) and young (14 dpi) mature nodules that show no senescence zone. In mature nodules, expression of the fusion is clearly visible in the infection zone as well as in the early nitrogen-fixing zone (ZIII) of the nodules (see Fig 3 in Tian *et al*. 2012 PNAS USA 109:6751). This is now stated in the discussion section (l. 364) as follows “*Noteworthy, signaling takes place in very young (7dpi) and in the nitrogen-fixing zone (ZIII) of 14dpi nodules (ref Tian et al. 2012), thus making it unlikely that signaling takes place during nodule senescence*.”

l. 377 Phosphorylation is one example (ref 42). Now indicated as “*Free RPs can be post-translationally modified (eg phosphorylated) or complexed...* ”

l. 387 The MDM2 name comes from Mouse double minute 2 homolog. More significantly, it is an ubiquitin ligase.

l. 410-411. References are now included “ *We have detected over the years signal 1 activity in nodules of Medicago plants grown under a variety of conditions (this study, [16,17,46]*.”

l. 414 Fix^-^ corrected

l. 423 The reference number is now included

l. 429 Rpf84 corrected. Rpf84 is a RPL22 protein, no homology to RPuL2 proteins.

l. 469-474 Strain construction used standard published procedures so anyone in the field should be able to follow the construction. “*The S. meliloti GMI12072 was constructed by introduction of the plasmid pGD2178 into a nsrA mutant strain (GMI12049,(17) by triparental mating using pRK600 as a helper plasmid. The S. meliloti GMI12071 was constructed by elimination of the gentamycin resistance marker of cyaK in GMI11556 (16) by marker exchange using the sacB selection procedure (56). Next, plasmids pGD2178 and pGMI50333 were introduced by triparental mating using pRK600 as a helper plasmid*.”

We have added the reference to S4 table as requested “*Strains genotype is described in S4 Table”* (l. 474).

l. 495 spaces included

l. 586 It is a commercial human antibody. Sigma Prestige Antibodies are tested for no/very low cross reactivity (see HPA050165 notice). There is no described cross reactivity of this antibody with other RPs.

l. 650 We are still waiting for Genbank accession number. We will communicate it to Plos One as soon as we get it.

l. -651 Computer programs are indicated “*The tree in Fig 3 was computed using* [*https://ngphylogeny.fr*](https://ngphylogeny.fr/) *(PhyML+SMS) (60), and visualized with Itol (61).”*

L 665 The references have been edited to PLOS One style.

Fig 1, 2, 4, 6 Miller units.

- In most cases (Fig 1BC, 2BCD, 4BC, 6) we used Miller units to compare the activity of the samples to the buffer control. Hence we show that the proteinase K treatment (Fig 1BC), the inactivation of the *cyaK* or *nsrA* genes in the reporter strain (Fig 2C, 4B) or the fiberglass treatment (Fig 2D, 4C) all reduced signal activity to buffer (background) levels. Similarly, we showed that the activity of purified ribosomes before RNAse treatment (Fig6) did not exceed background (Buffer) levels.
- They were two exceptions to this rule. In FIg 1A we compared the signal activity of different biological crude extracts. Then we standardized the activity to the amount (µg) of protein present in the sample. In Fig 5 we tested the activity of purified proteins of very different sizes (*ca* 1000 to 35 000 Da). Therefore we standardized the activity to the µM concentration of the protein to take into account size differences.
- The same rationale applies to SI figures.

Fig. 2 was modified as requested. Fig 2B is a single full purification experiment specifically performed for the publication (*ie* the three streptag-heparine-gel filtration steps have been performed in a row, no stop, no freezing in between). However, each individual purification step was optimized in preliminary assays. Fig 2C: error bars have been included as requested. Fig 2D is the result of a single experiment (note however that fiber-glass binding was demonstrated for the cognate *Medicago* protein (Fig 4C).

Fig 3 Panel A the resolution has been improved and the size of the text of the scale bar enhanced, as requested. All public accession numbers are shown. Accession numbers for the *Medicago* proteins are pending (revised annotation of sequences submitted to GenBank as indicated in the Material and methods section, l.651).

Fig 3B. We do not think it would be wise to include *E. coli* RPuL2 in this alignment (although it is very easy). As discussed above (reviewer #1), this alignment would reflect the phylogenetic relatedness of the *E. coli* and *Medicago* RPuL2 proteins without telling anything about the residues/motif important for signal activity. Instead, we propose in the paper (l. 396-403 and S7Fig) that basic residues of the RPuL2 proteins interact with surface-exposed acidic residues of the NsrA-receptor protein. In S7 Fig (that this reviewer could not review because the file was too large) we show the distribution of basic residues in both *E. coli* and *Medicago* RPuL2 proteins.

Fig 4A “kDa” added.

Fig 4BC: Error bars have been added as requested after incorporating a new experiment to the data set. The legend has been modified accordingly.

Fig5 Error bars have been added as requested. Because the tested peptides/proteins have very different sizes (*ca* from 1.000 da to 30.000 da), we cannnot express here activity/µg of protein. Activity/µM is better.

Fig 7 “FW” added as suggested in the title of the Y axis.

Fig 7B : the whole western blot picture is now shown. No degradation product was observed. See also the full-length image deposited at Plos One site.

End.
