# Supplementary material for: The ex planta signal activity of a Medicago ribosomal uL2 protein suggests a moonlighting role in controlling secondary rhizobial infection
Source: PLoS One. 2020 Oct 1;15(10):e0235446. doi: 10.1371/journal.pone.0235446 (PMC7529298; doi:10.1371/journal.pone.0235446)
Supplement: Attachment — Submitted filename: Responses to Reviewers Rev2.docx [file pone.0235446.s015.docx]

Sorroche *et al.*

**Reviewer#2**

**Fig 1**. To improve consistency, we have removed the cyaK and Buffer controls from panel C.

The amount of nodule extract (50 mg) is now indicated in the legend of Fig. 1B.

**Fig. 2**.

- “IV “was added back to panels A and B.

- Miller units is correct here. As shown in panel A, fractions IV 7 to 10 from the size exclusion column contain different amounts of RPul2 protein (which is the major protein in the sample at this stage of purification). We observed that the signal activity (Miller units, panel B) varies with the amount of RPuL2 protein present in the fractions (panel A).

- The fact that Fig 2B and 2D feature a single assay is now acknowledged at the end of the figure legend (l. 186): “ *Panels B and D feature the results of a single typical experiment.*”

- Fig 2D: The amount of purified RPuL2 protein used in this assay (1µg) is now indicated in the legend of fig. 2D (l. 185).

**Fig 4C**. Sorry, the figure was oversized. This has been corrected.

**l.240-243.** The specific activity of the *E. coli* RPuL2 protein was assessed independently in separate experiments, including fig 2C . This is now clarified l. 241 “ *The specific activity of the purified MtRPuL2A-strep protein was ca. 3-fold lower than that of E. coli RPuL2 (ca 10^5^ Miller units/µM) assessed in independent purification assays,…”*

End*.*
